# Gluing Living Bone Using a Biomimetic Bioadhesive: From Initial Cut to Final Healing

**DOI:** 10.3389/fbioe.2021.728042

**Published:** 2021-11-08

**Authors:** Philip Procter, Gry Hulsart-Billström, Antoine Alves, Michael Pujari-Palmer, David Wenner, Gerard Insley, Håkan Engqvist, Sune Larsson

**Affiliations:** ^1^ Department of Engineering Sciences, Division of Applied Material Science, Uppsala University, Uppsala, Sweden; ^2^ Biomimetic Innovations Ltd, Shannon, Ireland; ^3^ Department of Surgical Sciences, Division of Orthopaedics, Uppsala University, Uppsala, Sweden; ^4^ NAMSA, Chasse-sur-Rhône, France

**Keywords:** bone adhesive, biomechanical model, fracture healing, phosphoserine, calcium phosphate cement (CPC), orthobiologic, biomimetic

## Abstract

Osteoporotic fractures are a growing issue due to the increasing incidence of osteoporosis worldwide. High reoperation rates in osteoporotic fractures call for investigation into new methods in improving fixation of osteoporotic bones. In the present study, the strength of a recently developed bone bioadhesive, OsStic^tm^, was evaluated *in vivo* using a novel bone core assay in a murine animal model at 0, 3, 7, 14, 28, and 42 days. Histology and micro-CT were obtained at all time points, and the mean peak pull-out force was assessed on days 0–28. The adhesive provided immediate fixation to the bone core. The mean peak bone core pull-out force gradually decreased from 6.09 N (σ 1.77 N) at day 0 to a minimum of 3.09 N (σ 1.08 N) at day 7, recovering to 6.37 N (σ 4.18 N) by day 28. The corresponding fibrin (Tisseel) control mean peak bone core pull-out characteristic was 0.27 N (σ 0.27 N) at day 0, with an abrupt increase from 0.37 N (σ 0.28) at day 3, 6.39 N (σ 5.09 N) at day 7, and continuing to increase to 11.34 N (σ 6.5 N) by day 28. The bone cores failed either through core pull-out or by the cancellous part of the core fracturing. Overall, the adhesive does not interrupt healing with pathological changes or rapid resorption. Initially, the adhesive bonded the bone core to the femur, and over time, the adhesive was replaced by a vascularised bone of equivalent quality and quantity to the original bone. At the 42 day time point, 70% of the adhesive in the cancellous compartment and 50% in the cortical compartment had been replaced. The adhesive outwith the bone shell was metabolized by cells that are only removing the material excess with no ectopic bone formation. It is concluded that the adhesive is not a physical and biochemical barrier as the bone heals through the adhesive and is replaced by a normal bone tissue. This adhesive composition meets many of the clinical unmet needs expressed in the literature, and may, after further preclinical assessments, have potential in the repair of bone and osteochondral fragments.

## Introduction

The last 100 years have seen the development of clinical techniques that enable safe and effective surgical treatment of a wide range of orthopedic conditions. In particular, the application of medical imaging in combination with standard implants, capable of adequately fixing broken bones, has transformed clinical outcomes. However, the limits of standard fracture fixation implant performance have largely been reached, in particular, the ability of implants, such as plates, nails, and screws, to adequately maintain anatomical reduction and fixation in the bone of poor quality. In very complex fractures close to joints with osteochondral fragments, the number and size of fragments often make fixation of implant hardware impractical. This is either due to location, such as a joint surface (where a screw head would be undesirable), or to size as screws or pins would be too large to adequately fixate the fragments during surgery. These issues have led to the use of injectable bone void filler augmentation biomaterials in combination with fenestrated orthopedic screws with the aim of reducing the risk of screw cutout or migration where bone is either osteopenic or osteoporotic (Russell and Insley, 2017; Sallent et al., 2020). The dream orthopedic bone augmentation biomaterial would not only replace the missing bone but also immediately bond bone fragments to maintain reduction, enable rapid healing and bone remodeling, and reabsorb into the body at surgery. An effective bone bioglue, ideally using a biomimetic approach (mimics compounds and reactions that occur in nature), could potentially address unmet clinical needs in a range of orthopedic treatments. There are a number of excellent reviews (Heisse et al., 2006; Farrar 2012; Shah and Meislin, 2013; Bhagat and Becker, 2017; Böker et al., 2019; Sánchez-Fernández et al., 2019; Panagiotopoulou et al., 2021) that explore the range of properties that potential bone adhesive solutions should include. Broadly, adhesive candidates fall into two categories: non-biologically and biologically inspired. The former are frequently based on chemically engineered materials that have some attractive features and are tolerated by living cells (Granskog et al., 2018). The latter aim to use a more “bioinspired” adhesive strategy (Bré et al., 2013), and several of these have demonstrated promising preclinical evidence (Kirillova et al., 2018; Hulsart-Billström et al., 2020; Kirillova et al., 2020). However, further translation of adhesive candidates to clinical use is far beyond the resources of research teams, while medical device companies, who have adequate resources, view a bone adhesive as a high-risk investment as there is no bone glue predicate in clinical use.

Typically, osteochondral fragments are mostly cancellous bones, with some articular cartilage and potential cortical bone shell. The clinical goal is to restore the original position (anatomic reduction) and maintain this either through internal fixation with bone screws and a metal implant or through an external brace until healed. For a glue to achieve this result, it must immediately bond bone fragments at surgery and allow healing to occur through itself, eventually being completely replaced by a bone of equivalent quantity and quality. In fresh fractures, the fragment surfaces are wet and bloody, and fat is also present, which is a very challenging environment for adhesion. Additionally, cancellous bone healing is differentiated from cortical bone healing, which is thought primarily to be due to the greater local availability of certain cell factors that enable much faster healing and bony union (Han et al., 2015; Sandberg and Aspenberg, 2016). This begs the following questions: Would the same glue adhere equally well to both bone types and would the healing response be similar? Moreover, what would be an acceptable minimum initial strength for the different bone types in patients that may range from young and healthy to elderly and osteoporotic? This latter question is perhaps the hardest to answer as the literature reports a wide range of adhesive to bone bond strength values depending on the type of bone as well as bone condition and surface preparation. For the cancellous bone, a lower limit of 0.2 MPa has been suggested (Weber and Chapman, 1984), also a range of 0.5–1.0 MPa (Farrar 2012). Higher *in vitro* values are reported for bonding to the cortical bone, ranging from 3 MPa (Norton et al., 2020) to 9 MPa (Granskog et al., 2018). While greater strengths are in general desirable, the limiting factor will always be the weakest bone that is to be glued which in osteochondral bone fragments is the cancellous bone. The cancellous bone has relatively low tensile and shear strengths at low apparent densities, for example, dropping to ∼ 0.5MPa at an apparent density of 0.25 g/cm^3^ when trabecular orientation is longitudinal (Ford and Keaveny., 1996; Keaveny et al., 2001). For clinicians, key questions are as follows: (a) Does the adhesive bond bones effectively at surgery? (b) Is it sufficiently strong for the bone being bonded? (c) Will it keep the bone fragments in place until the bone itself can heal and fully assume this role?

The present study identified an adhesive formulation with a credible connection to human cell biology which is thought to increase the chances for such a glue to be biologically safe and effective. A key component is a nonessential amino acid, phosphoserine (PSer), that has already shown potential as a tissue glue component. PSer has been speculated to be part of a reversible bonding mechanism in bones (Hansma et al., 2005), while fractured trabecular bone surfaces have demonstrated high concentrations of phosphoserine (Thurner et al., 2009). A modified lipid form, phosphatidylserine, has been shown to be a factor in cadherin–lipid linking as well as signaling pathways (Yap et al., 2015), and has also been recognized as part of a very effective hydroxyapatite binding mechanism (Merolli and Santin, 2009). A surprising discovery was that PSer, used with fine particulate calcium, enabled bonding effects to be obtained in both calcified and uncalcified tissues (Pujari-Palmer et al., 2018 and Liu et al., 2019, respectively). This is suggestive of a common cell adhesive mechanism that is in some way enabled by the amino acid. PSer-modified calcium cement was demonstrated to enhance strength, increase bioactivity, and rapidly remodel in animal models (Reinstorf et al., 2006; Mai et al., 2008). More recently, collagen/nanohydroxyapatite scaffolds that are modified with phosphorylated amino acid (O-phospho-L-serine–OPS) to mimic bone tissues and induce cell differentiation have been developed (Salgado et al., 2019) The preceding literature adds weight to the idea that phosphoserine and phosphoserine/calcium-based biomaterials play a biomimetic role in the calcified tissue void filling and adhesion. PSer in combination with tetracalcium phosphate (TTCP) was shown to have good long-term osseointegration and bioresorbability at 52 weeks in a lapine model (Kirillova et al., 2018). The same cement used to fill cranial defects in sheep demonstrated new bone formation and increased strength at 12 weeks, and further osseoinegration and strength *versus* control at 1 and 2 year follow-up (Foley et al., 2020). In a study of simulated canine mandible repair procedures, while control (unfractured bone) was strongest in mechanical testing, the fixation with TTCP + PSer glue showed promise with greater strength than dental wire when either was combined with dental composite (Geddes et al., 2020). Dental implant fixation with TTCP + PSer was further explored in a canine *in vivo* oversize osteotomy model with 3.3 mm Ø implants glued into a 5.5 mm Ø osteotomy (Cochran et al. 2020). The mean removal torques were reported to be 22.2, 45.7, and 104.7 cmN at 24 h, 10 days, and 4 months, respectively. Norton et al. (2020) reviewed preclinical model data for the TTCP+PSer adhesive and considered the properties that would qualify “bone glue” in the repair of simple and comminuted fractures. However, no published data that support the *in vivo* efficacy of a bone adhesive in cancellous bone repair have so far been presented over a clinically relevant time frame. Indeed, there are few animal models at all that enable an assessment of strength measures, particularly in the healing of the cancellous bone (Sandberg and Aspenberg., 2016).

In developing an optimal adhesive formulation for bone, a number of different forms of calcium were evaluated including silicates, octacalcium phosphate, tetracalcium phosphate, and alpha/beta tricalcium phosphates, all of which perform well in a range of clinical applications (Habracken et al., 2016). In the present formulation, a particular PSer αTCP and calcium *meta*-silicate (also known as wollastonite) combination was found to be favorable for small bone fragment fixation. The calcium *meta*-silicate was added to enhance the setting reaction strength and beneficially influence both bone healing and osseointegration (Sanmartin de Almeida et al., 2018). Where the bone fragments are well reduced, αTCP gave higher values of shear resistance, up to 6 MPa, than TTCP. To win the surgeon more working time before the adhesive sets, a retardant may be added, and the αTCP formulation gave higher shear strengths (>2 MPa) with higher retardant concentrations (Pujari-Palmer et al., 2020). The αTCP formulation chemistry is favorable as this sets *via* a classic acid–base reaction, where PSer is an acidic molecule and αTCP is a basic calcium salt, similar to CPC cement. The setting reaction chemistry is not similar in which the αTCP does not transform to hydroxyapatite and is maintained in a metastable form releasing calcium ions to the reaction (Pujari Palmer et al., 2018). Additionally, αTCP has significantly higher solubility under low pH conditions than other calcium salts such as, βTCP and octacalcium phosphate (Suzuki and Insley., 2019), making it a good choice to drive the reaction with PSer. Moreover, αTCP has an extensive clinical use history as an active bone void filler and has a higher remodeling rate than hydroxyapatite (Carrodeguas and De Aza, 2011; Eliaz and Metoki., 2017). The calcium *meta-*silicate was added to enhance the setting reaction strength and beneficially influence both bone healing and osseointegration (Sanmartin de Almeida et al., 2018).

To evaluate the biological safety of PSer αTCP adhesive formulations, a standard subcutaneous murine model was used to evaluate these at 6 and 12 weeks (Hulsart-Billström et al., 2020). The adhesive formulations evaluated proved to be safe both on histological and gene expressional levels, with no changes in the regulation of immune and bone marker genes. The histological analysis showed no influence on the surrounding connective tissue, indicating a biocompatible nature of the adhesive. For assessing the mechanical strength of the glued bone, it was already noted (Sandberg and Aspenberg., 2016 vide supra) that there are very few animal models that are able to do this, particularly in the cancellous bone. Keller et al. (1985) created a standardized osteochondral fracture model in canine knees in which a cylindrical section was chiseled out, creating a wedge section. The bone cylinder wedges were subsequently either glued back in place with fibrin or fixed with Kirschner wires. The bone cores/knees were then explanted after sacrifice of the animal and subjected to tensile testing in which the fibrin showed a 7-fold increase in pull-out strength by day 4 and further increase by day 7. It was concluded that the fibrin sealant can be used as an alternative method for the fixation of small, well-adapted, osteochondral fragments, provided reliable immobilization is obtained. Kirillova et al. (2020) presented a rabbit distal tibial osteotomy model, where primary stability was obtained by screws in combination with an adhesive and then tested to failure in shear with the screws removed. Cochran et al. (2020) reported a canine dental grafting model in which the adhesive was used to obtain primary stabilization of dental implants in an enlarged osteotomy site. The dependence of interindividual biomechanical properties on a well-developed biomechanical testing methodology was emphasized by Prodinger et al. (2018) who studied bending testing to failure in rat femurs. They showed that precision of the method affected the statistical power of the study, and by adapting the biomechanical testing, interindividual variation could be reduced. This is relevant to the models developed by the present authors.

**FIGURE 1:**
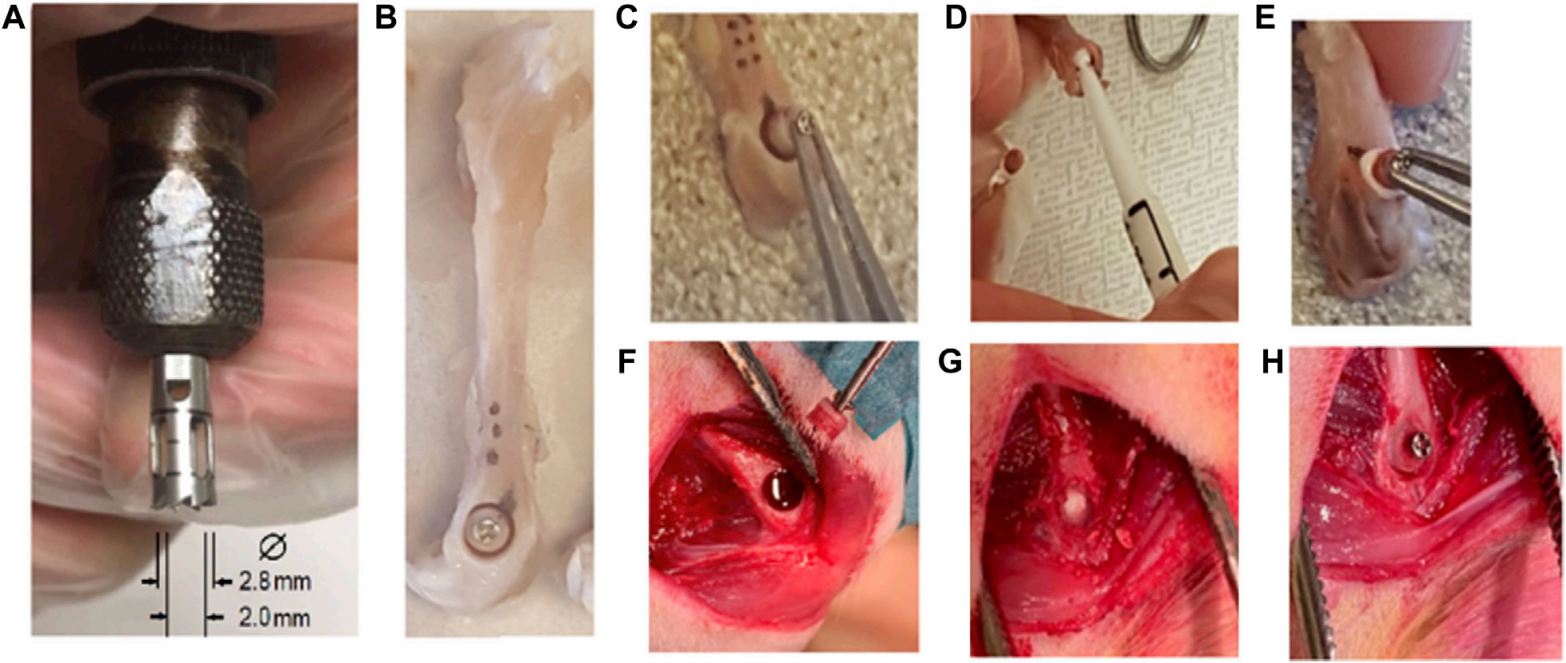
*Ex vivo* and *in vivo* methods for the bone core osteotomy and gluing procedure. *Ex vivo*: **(A)** bone core trephine tool, **(B)** murine femur extraction screw placed and bone core cut, **(C)** removal of bone core, **(D)** placing adhesive in the osteotomy, and **(E)** bone core replaced and glued in place. *In vivo*: **(F)** blood flow after bone core removal, **(G)** additional adhesive layer at the bottom of bone core osteotomy, and **(H)** bone core replaced and glued in place.

In the absence of a published animal model with which to evaluate adhesive effects, an *ex vivo* model was developed (Procter, 2019) and then evaluated *in vivo* in the present study. The model (see Figures 1A-E) is based on a cortical/cancellous bone core harvested from the lateral aspect of the distal femur in the rat. The core, fitted with a small screw and applied with tensile axial loading, was then glued back in place either with Tisseel, a commercially available medical grade fibrin glue, or with the PSer-based adhesive, OsStic. It should be noted that fibrin glue is only approved as an hemostatic tissue sealant rather than a hard tissue adhesive. Fibrin was chosen as the control in the present study as it has been frequently used in orthopedic preclinical studies that explore adhesion of bone fragments (Plaga et al., 1992; Keller et al., 1985). The amount of adhesive in the layer between the bone core and the outer diameter of the trephine cut was estimated^1^ at ∼9 mm^3^ which assumes that the core is replaced concentrically in the osteotomy bone. The subsequent *ex vivo* study results showed that Tisseel weakly bonded the metaphyseal bone cores, while OsStic produced > 30-fold higher mean peak forces at failure (7.64 vs 0.21 N). The failure modes were consistently disparate, with Tisseel failing gradually, while OsStic failed abruptly, as would be expected with a calcium-based material. Imaging of the bone–adhesive interface with microcomputed tomography revealed that failure occurred more often within the cancellous bone (75% of tested samples) rather than at the adhesive interface. In translating this method to an *in vivo* study, it was expected that the *ex vivo* results could be replicated as the rat distal femur was recommended as a biomaterial test site by Li et al. (2015). This advice neglects that there is usually very late physeal closure in the rat (>52 weeks) and a non-solid paste-like biomaterial, such as an injectable cement, will be subjected to a very wet bloody field as blood escapes from the drilled hole. A technique whereby the bottom of the core hole was sealed by a first layer adhesive was developed, with there being no strength loss in layering the adhesive (see Figures 1F-H). Subsequently, bone core pull-out strengths comparable to the *ex vivo* results (Procter et al., 2019) were then obtained, and the adhesive layering technique was adopted for the remainder of the *in vivo* studies.

In conclusion, a bioadhesive based on PSer, αTCP, and calcium *meta*-silicate has been developed and already evaluated *ex vivo* in a novel murine model bone core model that, after some further development, is now sufficient to undertake the *in vivo* evaluation that is presented herein. The aim of the present study was to delineate the *in vivo* characteristics of the bioadhesive by determining the following: 1) the strength of the glue over time from the point of surgery, 2) the bone–adhesive relationship during healing, and 3) whether the bioadhesive addresses unmet clinical needs.

## Methods

### Animal Model and Surgical Procedure

The *ex vivo* bone core model, developed using male Sprague–Dawley rats as a precursor to this *in vivo* study (Procter, 2019), was submitted to the local ethical committee as the basis for the methodology in the following procedure. The animal study was approved by the Uppsala Committee of Animal Research Ethics (5.8.18–09,216/2018) according to the Federation of European Laboratory Animal Science Association’s guidelines. To allow paired observations, each animal received bilaterally either the test article OsStic adhesive or the control Tisseel® (Baxter Medical AB, Kista, Sweden). Male Sprague–Dawley rats (250–320 g) were randomized into five time-points (days 0, 3, 7, 14, and 28) (n = 8). The animals were anesthetized in an induction chamber with 0.45l/ min air and 4% isoflurane, and later placed on a 37°C heat pad and a facemask with 0.45l/ min air and 1.5–2.5% isoflurane (Isoba vet®, Schering-Plough, United States). Buprenorphine (0.05 mg/kg) was administered subcutaneously preoperative, and 1 ml sterile saline (Fresenius Kabi, Uppsala, Sweden) was administered perioperative to prevent dehydration. The rat thighs were shaved and washed three times with chlorhexidine ethanol (5 mg/ml) (Fresenius Kabi, Uppsala, Sweden). Local anesthesia 2.5 mg/ml bupivacaine + 5 µm/ml adrenaline (Marcain®, Aspen Pharma, Dublin Ireland) was applied topically prior to a longitudinal lateral skin incision followed by blunt dissection between the m. vastus lateralis and m. biceps femoris, with local anesthesia applied dropwise. A metaphyseal fragment was created by drilling using an irrigated (Saline, Fresenius Kabi, Uppsala, Sweden) trephine (outer Ø2.8 mm, inner Ø2.0 mm, Dental mind, Gothenburg, Sweden), to an approximate depth of 2 mm. A pilot hole was drilled using a Ø0.75 mm drill bit (Dormer Pramet, Halmstad, Sweden), and a steel screw (Micronwings Screws Self Tapping Ø1.0 mm × 2 mm Pan Head 304 Stainless Steel) was manually inserted using a forceps and a miniature precision screwdriver with a tip size of 1.2 mm. After inserting the screw to a depth of 1.5 mm, the osseous fragment was gently displaced, and approximately 0.2 ml Tisseel was injected into the defect site through the custom syringe provided as a part of the Tisseel kit. For OsStic application, an approximate volume of 0.1 ml was applied using a spatula to still the blood flow. The delivery was completed within 1 min 10 s, and the material was allowed to set for 6 min, after which a second batch of 0.1 ml OsStic was applied on the osseous fragment that was repositioned within 1 min 40 s into the metaphyseal defect. The wound was closed subcutaneously and inversed transcutaneously (Vicryl^®^, 4–0, C-3 needle, 45 cm, Ethicon, Johnson & Johnson, Somerville, United States). Buprenorphine (0.05 mg/kg) was administered subcutaneously three times daily for 3 days for analgesia (Temgesic®, Sheringer Plough Brussels, Belgium). Free load bearing and activity were allowed. At days 0, 3, 7, 14, and 28, the rats were anesthetized in 4% isoflurane and euthanized in a CO_2_ chamber (Makrolon® III cage with dimensions 382 × 220 × 150 mm). The cycle of the CO_2_ chamber was 50% oxygen and 50% CO_2_ for 6 min, followed by 100% CO_2_ for 6 min and then a second cycle of 2 × 6 min of CO_2_, after which the rats were confirmed dead by decapitation, and both femurs were then explanted.

### Longitudinal Study

A further separate group of 6 animals were included in the study in which µCT examinations were made weekly over 6 weeks and histology performed at the 42 day point of sacrifice. The goal of this study group was to look for any correlations with the study detailed in the *Animal Study Surgery* section to see if this would give insights into the adhesive performance that would ordinarily need a much larger animal study. This could potentially enable reduction and refinement of the animal model. This group did not have screws implanted; otherwise, it had identical bilateral implantations of bone cores one each of OsStic and fibrin glue as described below. These animals were scanned weekly where they were anesthetized in an induction chamber with 0.45l/ min air and 4% isoflurane and transferred to a gantry bed heated through hot air to prevent hypothermia (isoflurane 1.0–2.5% and air at 450 ml/min). Each femur was scanned using source voltage: 90 kVp; current: 150 mA; pixel size: 36 μm; filter: 0.1 mm^3^; exposure time: 150 ms; frame averaging: 1; rotation step: 0.70; and field of view: 68 mm. At day 42 , the animals were sacrificed, and histological slides were subsequently prepared.

### Mechanical Testing

**FIGURE 2:**
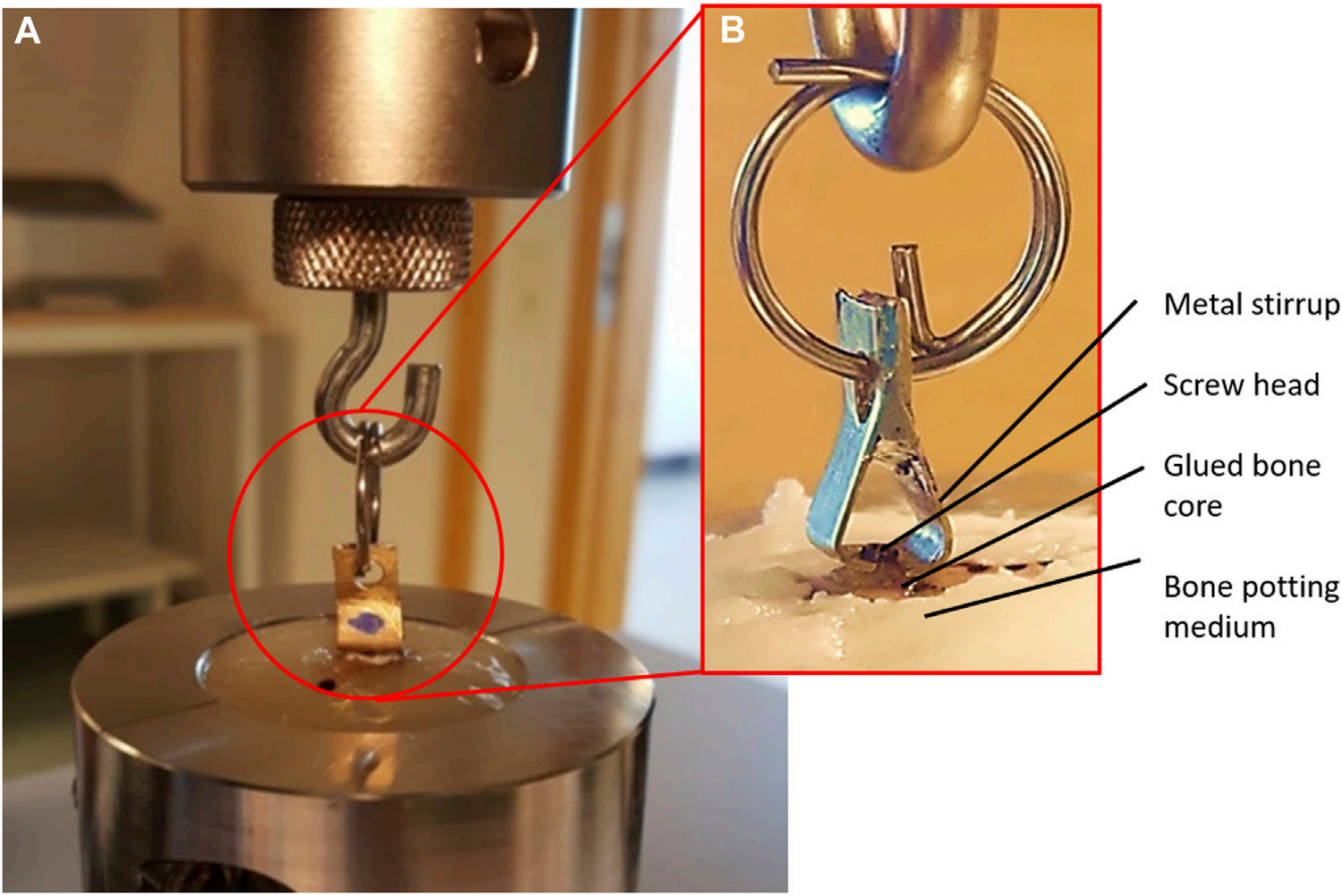
**(A)** Tensile test setup and **(B)** Detail of loading jig.

The murine bone core model testing protocol used in this article was initially developed (Procter, 2019) for the assessment of the *ex vivo* adhesive force of OsStic and fibrin Glue. The testing in this earlier study was undertaken on femurs excised from freshly killed animals and then frozen for later evaluation. It was decided to add a day 0 animal group in the present study to validate the immediate strength and compare with the earlier *ex vivo* data to see if storing the adhesives in freezing temperatures had degraded or altered the performance of the adhesives used. The same preparation and testing protocol were followed in the present study in which each femur was potted in resin to enable pull-out testing of the bone core. Following sacrifice, the excised femurs were carefully cleaned taking care not to disturb the bone core implantation site. Any excess adhesive that prevented easy attachment of the tensile test and jig was carefully removed using a sharp-tipped scalpel blade. Each femur was truncated using a diamond-bladed bandsaw (IMEB Inc., United States ) in the diaphyseal region, to fit the size of the potting mould, and was potted as a 50% (wt%) mixture of Bostik Epoxy Rapid and calcium phosphate [8 g (g) epoxy and 8 g of calcium phosphate]. During potting, rodent femurs were oriented using a screw inserted into the metaphyseal plug, perpendicular to the potting surface. The epoxy was allowed to cure for 4 h at 37°C, in humidity, throughout the curing process. The exotherm of the epoxy was monitored using a thermocouple (Omega JMTSS M050G-150) and a temperature input module (National Instrument NI9211), with measurements taken every second, to ensure the curing temperature remained below 40°C. Approximately 16 g of potting epoxy prepared was used for each femur, in an 18 cc cup [Ø 3.8 cm (cm)]. The curing temperature was monitored when different amounts of epoxy were replaced with calcium phosphates, in 4 g increments. The following compositions were tested: 16 g of epoxy, 12 g of epoxy with 4 g of calcium phosphate, and 8 g of epoxy with 8 g of calcium phosphate. A custom-designed loading jig was placed under the screw and preloaded to 0.5 N, as part of a tensile testing rig (see Figure 2).

The screw and osseous fragment were then displaced (tensile loading) at a rate of 1 mm/min on a Shimadzu AGS-X mechanical testing machine, equipped using a 50 N load cell (#SM-50N-168, Shimadzu Europa, Force version 1.01, Shimadzu Europa). The adhesive stiffness was estimated from the best fit line to the linear part of the loading curve. The peak pull-out force value was determined from the force displacement data, and the mean of these peaks at each time point was calculated (mean peak pull-out force is hereafter referred to as *mppf*). The *mppf* was also estimated for the Tisseel force displacement curves.

### μCT

The explanted femurs were examined after the mechanical testing by μCT (SkyScan 1,176, Kontich Belgium) using source voltage: 50 kVp; current: 499 mA; pixel size: 8.87 μm; filter: 0.5 mm aluminum; exposure time: 1,000 ms; frame averaging: 4; rotation step: 0.37; and field of view: 68 mm. The NRecon software was used for reconstruction. The software CTAn was employed for the analysis, while CTvox was applied for bone imaging; all pieces of software were from SkyScan, Bruker MicroCT, Kontich, Belgium.

### Histological Preparation and Analysis

The explanted femurs were dehydrated in alcohol solutions of increasing concentration (60, 80, 95%, and 2 × 99.9%) (ethanol, TechniSolve, VWR, Solna, Sweden) and later infiltrated in increasing concentration of Technovit 7200 (Kulzer Exakt, Histolab, Gothenburg, Sweden) mixed with 99.9% ethanol (30:70, 50:50, 70.30, 100%, and 100%). For each femur, one transverse sagittal cross section was obtained using a micro-cutting and micro-grinding system (EXAKT System, Advanced Technologies GmbH, Germany), with the thickness of each section ranging between 10 and 46 µm. The sections were stained with modified Paragon for qualitative analysis (staining and section reading performed by NAMSA, Chasse Sur Rhone, France).

## Results

### Animal Study Surgery

The OsStic and Tisseel arrested the hemorrhage from the bone core osteotomy defects, which improved the healing by preventing the large hematoma formation at the incision site. All animals recovered quickly and immediately used their hind limbs after surgery and were standing upright and expressing natural behavior the day after surgery.

### Histology

**FIGURE 3:**
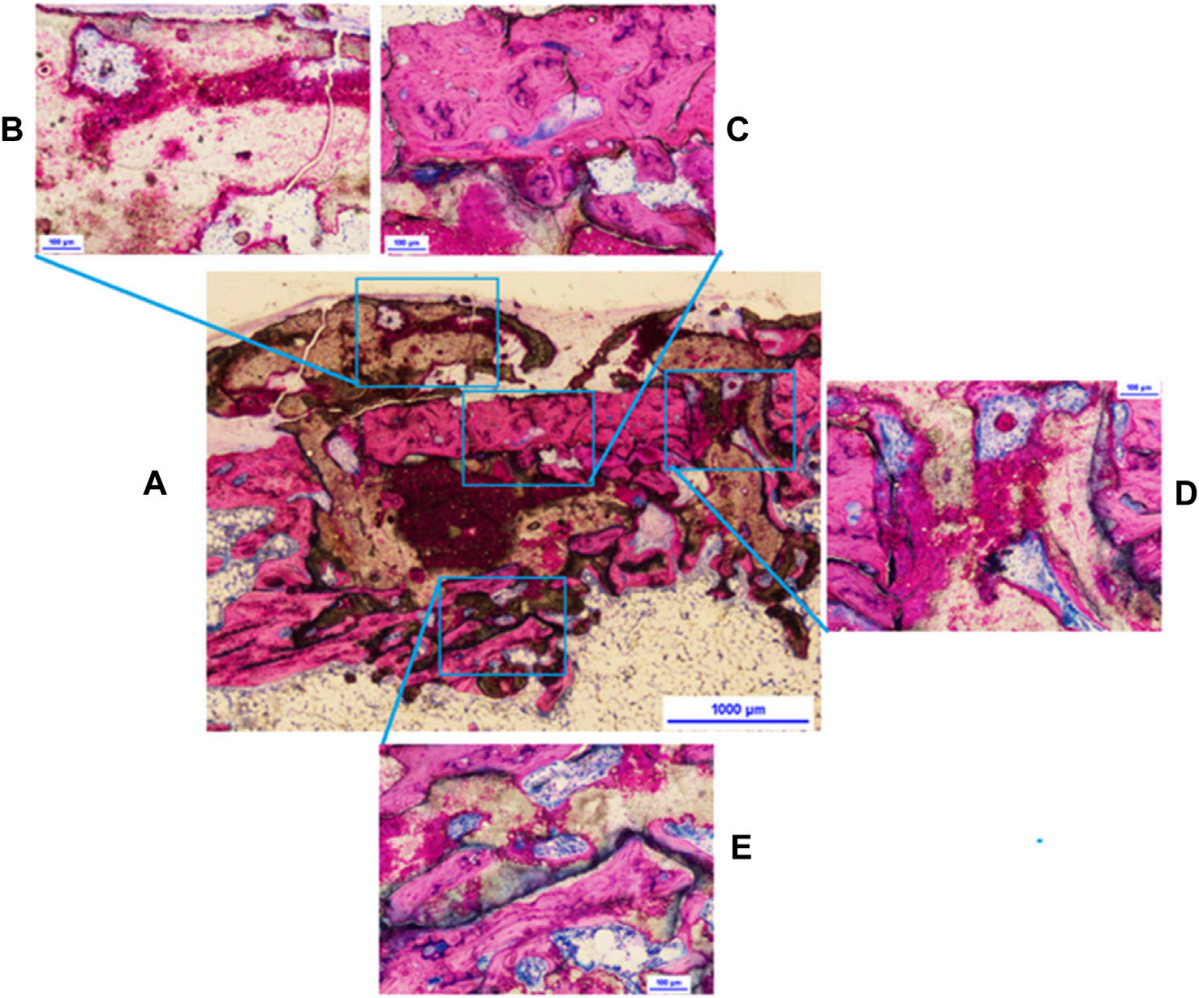
Adhesive at 42 days: **(A)** overview of the bone core, **(B)** active resorption of adhesive excess with vessels visible inside resorption cavities, **(C)** resorption of the bone core, **(D)** interface between core **(to the left)** and host living cortical bone, BMUs throughout the material, and **(E)** evidence of adhesive material remodeling, lamellar trabeculae.

**FIGURE 4:**
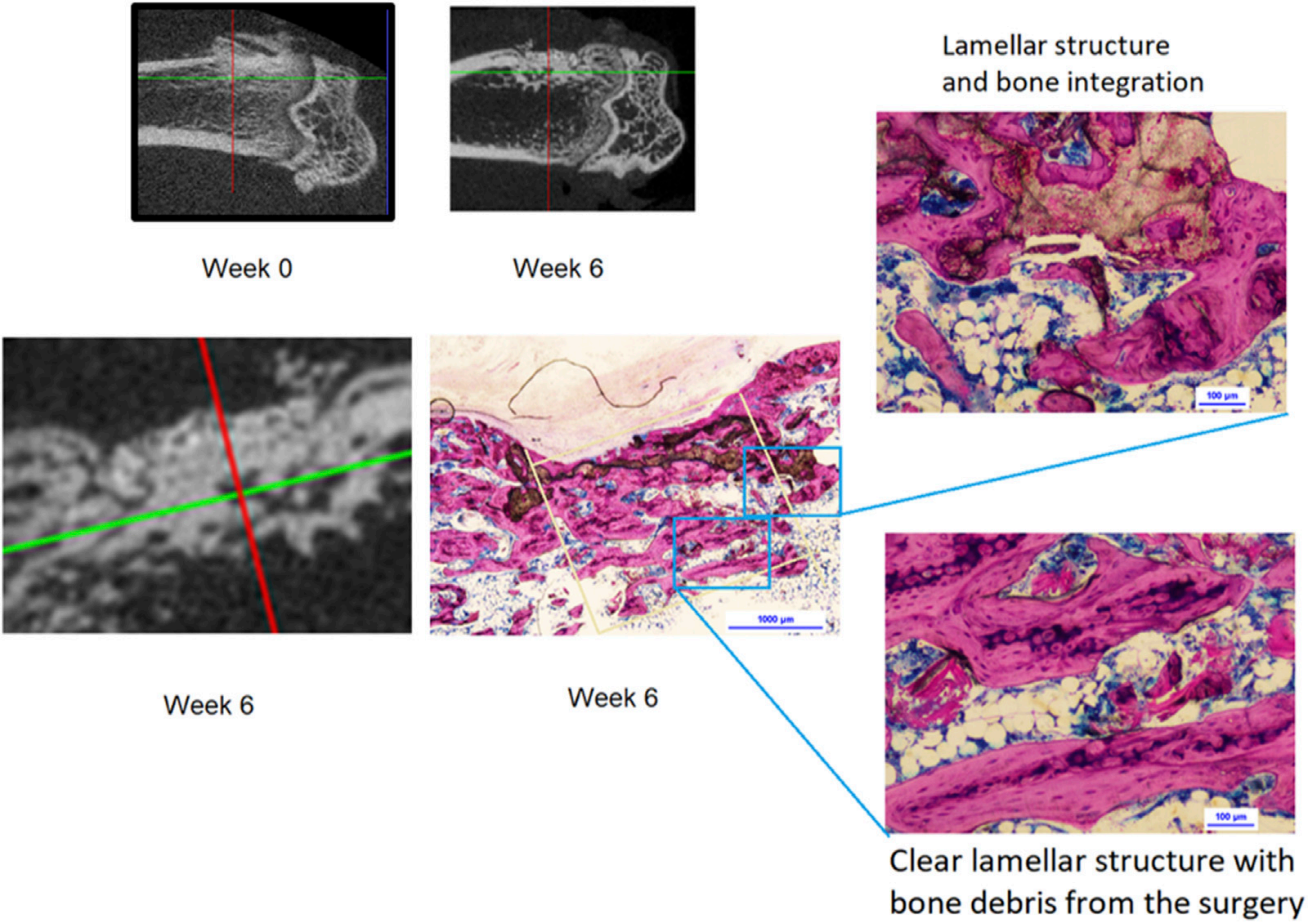
Micro-CT images at weeks 0 and 6; 42 day histology shows complete bone layer fully covering the adhesive material following the cortical line. Material is fully integrated in the newly formed bone showing early formation of the osteonal structure.

The histologic observational findings (n = 3/group/time) were evaluated from days 3 to 42 by day and by material, Tisseel or adhesive. A summary of the histological results, for each time point, can be viewed in Supplementary Tables S1–S4. The micrographs were selected to illustrate the most meaningful findings in each group, and it should be noted that while they were not presented on the same scale in the two groups, the magnification bars at each time point have the same numerical value to aid comparison. The summary of findings is as follows: at day 3, the adhesive had greater proliferation and cell differentiation precursor events than the Tisseel. At day 7, the adhesive also had greater early osteogenic activity than Tisseel. By day 14, the biodegradation, led by multinucleated cells, resulted in 3D porous scaffolding of the adhesive, and there was appositional bone growth. At day 28, healing started within the cortical compartment with around 10% of the adhesive material biodegraded. In the cancellous compartment, ongoing active healing of the adhesive was greater than that of the Tisseel group, with approximately 40% adhesive degradation. At day 42 (detailed views in Figures 3, 4), the cortical bone plug autograft was maintained in position and osseointegrated. There was controlled bone repair as no bone outgrowth was observed despite an excess of adhesive outside the bone envelope. In the cortical bone compartment, ongoing healing was observed with approximately 50% adhesive biodegraded. In the cancellous compartment, bone maturation was greater in Tisseel than in the adhesive group, with approximately 70% of the adhesive biodegraded. In conclusion, the adhesive material acted as a multifunctional biomimetic osseous matrix. It showed adhesive, bioactivity, osseointegration, osteoconduction, and biodegradability properties without inducing signs of local adverse effects or uncontrolled bone repair when applied at the cortical or cancellous bone defects in the present rat model.

### Mechanical Testing

**FIGURE 5:**
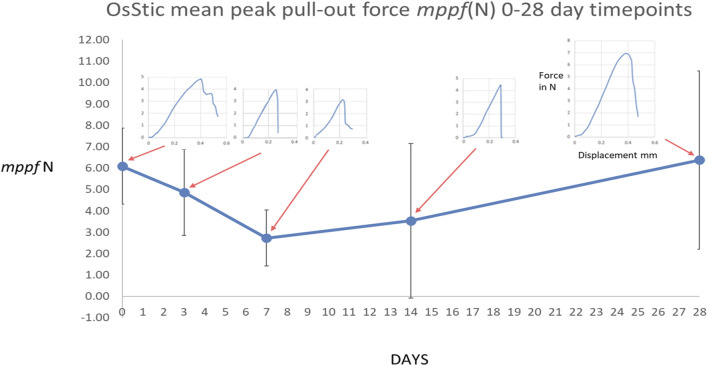
Mean peak pull-out force for the OsStic adhesive with representative force displacement curves at each time point (selected a curve closest to the *mppf*).

**FIGURE 6:**
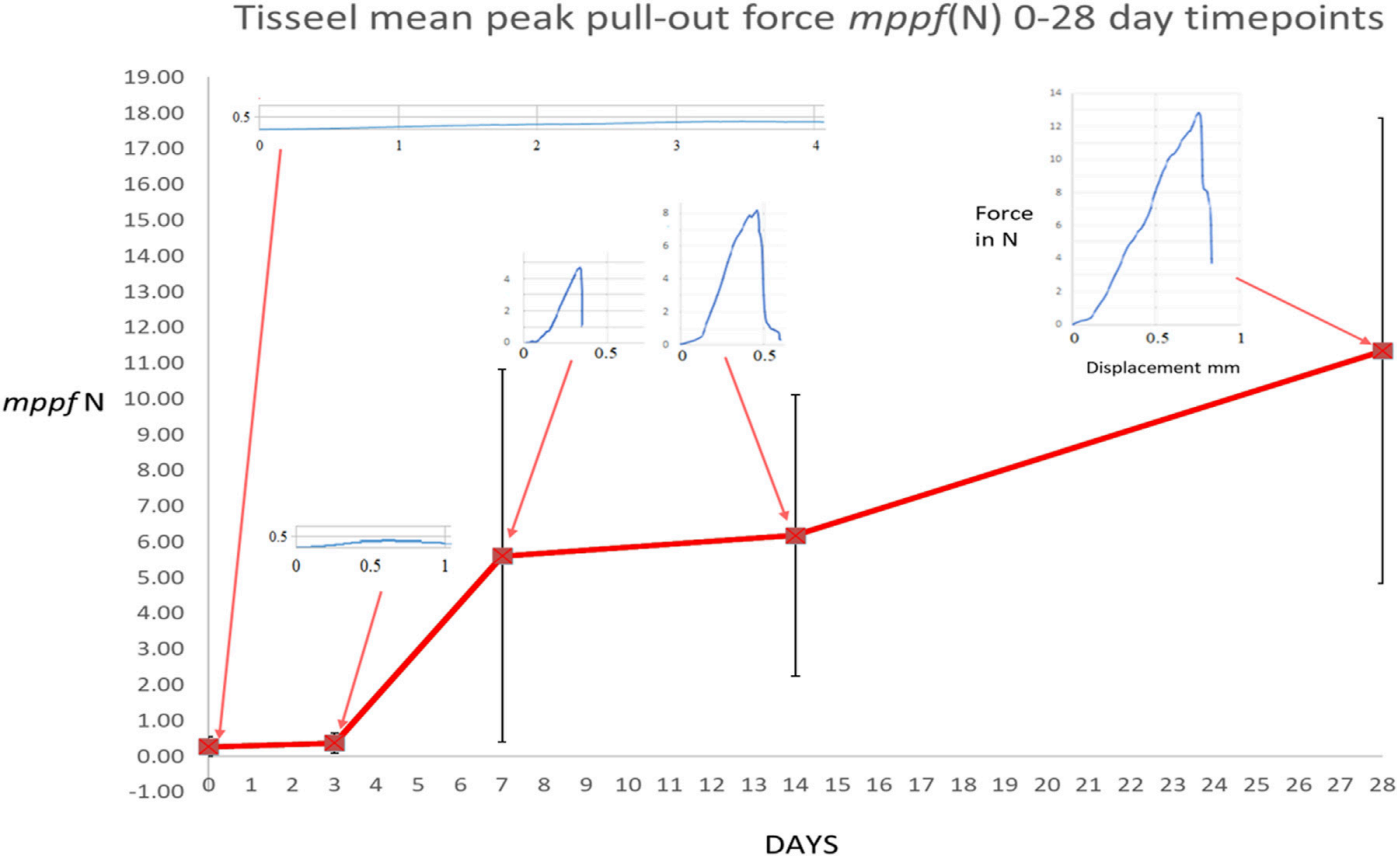
Mean peak pull-out force for the Tisseel control with representative force displacement curves at each time point (selected a curve closest to the *mppf*). The OsStic curve is included for comparison.

**FIGURE 7:**
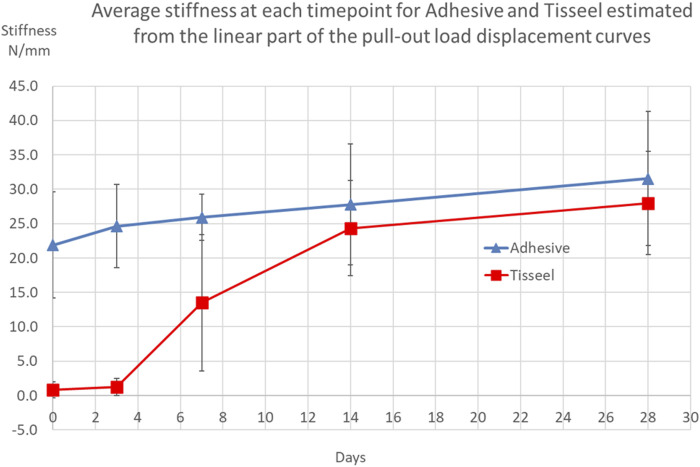
Average stiffness taken from load displacement tests for each material at each time point.

The peak bone core pull-out force was obtained from the electronic record of each bone core pull-out test for both OsStic and Tisseel at each time point with the *mppf*, standard deviation, and number of samples recorded (see Table 1). The *mppf* for the OsStic adhesive with representative force displacement curves at each time point (selected a curve closest to the *mppf*) is plotted in Figure 5. The Tisseel control *mppf*, at each time point, is plotted separately in Figure 6 together with representative force time curves. Comparing the fibrin and OsStic adhesive time course, the differences in *mppf* between the materials were significant at days zero and 3 (*p* < 0.001), while there was no significant difference at the later 3 time points. The OsStic adhesive *mppf* curve decreased to a minimum at day 7 that was half the initial value, and then steadily increased in *mppf* through day 14 to regain its initial value by day 28. The Tisseel by comparison was very low at days 0 and 3 and then abruptly increased to equal the day 0 OsStic adhesive level at day 7, and continued to increase steadily at 14 and 28 days. The *mppf* of each material was significantly different at both day 0 and day 3 with OsStic being, respectively, 23 and 13 times greater than that of the Tisseel. At the 7, 14, and 28 day time points, the Tisseel mppf values were, respectively, 2, 1.7, and 1.8 greater than those in the OsStic group, although these values were not significantly different due to the large variances. The overall form of the *mppf* curve was similar in both materials at days 7, 14, and 28. The representative curves for OsStic in Figure 5 are very similar, showing an initial nonlinear portion as the axial load is applied, and then a linear region usually followed by an abrupt failure in the range 0.2–0.4 mm displacement. In contrast, Tisseel, in Figure 6, has a quite different characteristic at days 0 and 3 typically showing large displacements at failure, respectively, 5+ mm and 1+ mm, and very low maximum values. Usually, the Tisseel remained attached to the bone and the bone core even when the core was quite separated from the main portion of the bone. Then from day 7 onward, there was an abrupt change in behavior with much larger failure loads, and the load displacement mirroring the adhesive in form showing an initial nonlinear portion as the axial load is applied, and then a linear region usually followed by an abrupt failure in a similar range 0.2–0.4 mm. The linear portion of each load displacement curve was used to estimate stiffness which was averaged for each material at each time point (see Table 1). The adhesive shows an approximately linear increase in mean stiffness over the test period (see Figure 7), and the mean stiffness was significantly higher than that of the Tisseel at days 0, 3, and 7. The Tisseel had low stiffness at days 0 and 3 and increased by day 7, and then continued at the level of the adhesive at days 14 and 28.

**TABLE 1:** Adhesive and Tisseel mean peak pull-out force (*mppf*) and mean stiffness at each time point.

	Adhesive					Tisseel					Adhesive vs. Tisseel	
Day	Samples, n	*mppf* N	Std. dev, N	Mean stiffness, N/ mm	Std. dev, N/ mm	Samples, n	*mppf* N	Std. dev, N	Mean stiffness, N/ mm	Std. dev, N/ mm	Difference in *mppf p* value	Difference in mean stiffness, *p* value
0	8	6.09	1.77	21.89	7.70	6^a^	0.27	0.27	0.85	1.15	<0.0001	<0.0001
3	8	4.86	2.01	24.63	6.07	8	0.37	0.28	1.24	1.24	<0.0001	<0.0001
7	6^a^	2.73	1.31	25.92	3.35	7^a^	5.61	5.21	13.50	9.91	0.1893	0.0141
14	8	3.53	3.62	27.79	8.77	8	6.18	3.94	24.34	6.95	0.2716	0.3979
28	8	6.37	4.18	31.56	9.72	4^b^	11.34	6.50	28.00	7.52	0.1350	0.5380

a unable to test all 8 animals due to screw misplacement/damage during retrieval .

b 4 screws that failed by stripping were excluded from the bone core failure group.

### Failure Modes

**FIGURE 8:**
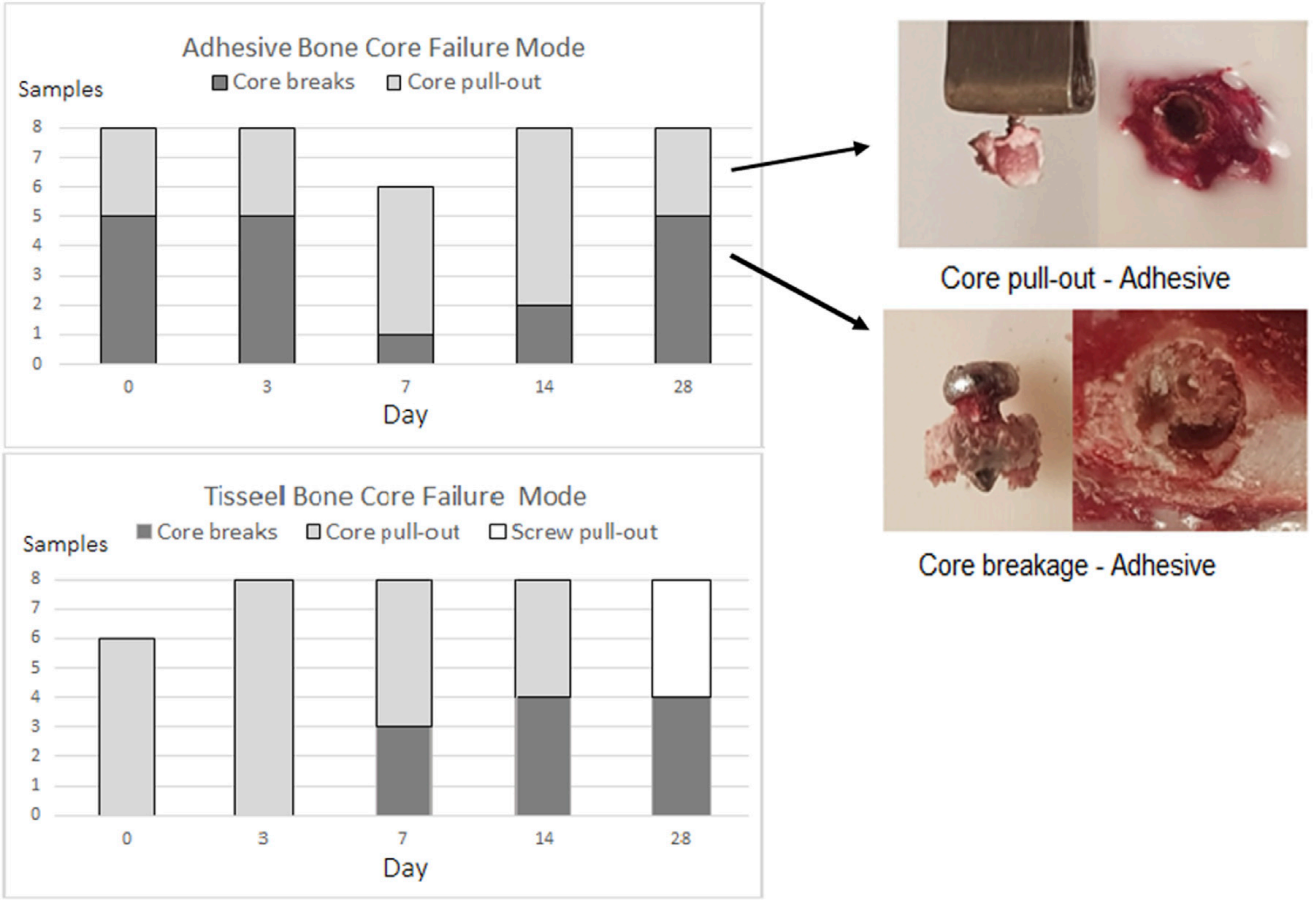
OsStic adhesive and Tisseel bone core failure modes.

Three failure modes were observed in the Tisseel group: bone core pull-out, bone core breakage, and screw stripping, whereas only the first two modes were seen in the adhesive group, and these are summarized in Figure 8. The failure mode in each test sample was visually assessed both from digital images and from the microCT scans. Two observers assessed these images independently and then compared findings to reduce subjective errors. Adhesive bone core breakage occurred mostly at days 0, 3, and 28 with the minimum of 1/6 samples at day 7. Further analyzing all adhesive broken cores, breakage was associated with a significantly higher (*p* < 0.0001) *mppf* of 6.79 N (σ 3.01 N) compared with all cores that failed in pull-out where the mppf was 3.01 N (σ 1.77 N).

### Under Bone Core Volume of Interest and Force Correlation

In the present study, bone density scans were not taken before surgery and were taken only after implantation. Because the radiological density of the adhesive is so similar to that of bone, it was not possible to define a meaningful postoperative bone density at the adhesive–bone core interface. As bone volume (BV/TV) may be considered as equivalent to apparent density/tissue density (Adams et al., 2018), it was decided to use this as a proxy for bone density, and determine this for bone adjacent to the adhesive/bone core site.

**FIGURE 9:**
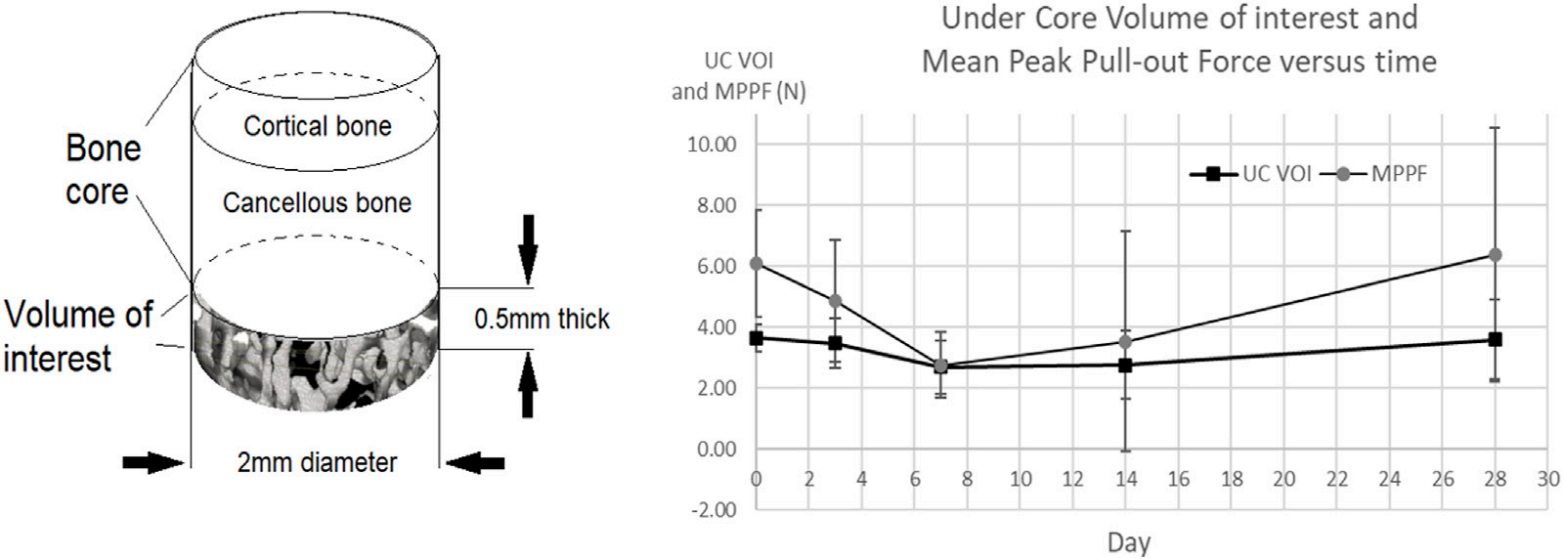
Under the core volume of interest mean value at each time point (UC VOI) *versus mppf* for the OsStic adhesive.

To test whether or not there was a relationship between the bone volume (BV/TV) adjacent to the adhesive and the mppf at each time point, a cylindrical volume of interest under each bone core (UC VOI) was defined and the bone volume (BV/TV) output from analysis of the CT scan images (see Table 2; Figure 9). The overall form of the UC VOI bone volume curve was similar to the mppf, having a minimum around days 7–14 and maxima at days 0 and 28. The range and values of BV/TV measured are within those reported in the literature for male Sprague–Dawley rats (Matsushima et al., 2001; Tajul Ariff et al., 2012). The mppf and UC-VOI were found to be strongly correlated at 0.89 correlation coefficient (Excel Pearson test).

**TABLE 2:** Under the core volume of interest (UC-VOI) mean value *versus mppf* (N) for the OsStic adhesive at each time point.

		Adhesive		UC-VOI	
Day	n	*mppf* (N)	SD (N)	Mean BV/TV	SD
0	8	6.09	1.77	3.66	0.45
3	8	4.86	2.01	3.47	0.80
7	6	3.09	1.08	2.69	0.88
14	8	3.53	3.62	2.76	1.12
28	8	6.37	4.18	3.59	1.31

## Discussion

This study represents the first *in vivo* bone core model, modified from the previously reported *ex vivo* bone core model (Procter, 2019). This was carried out to limit the copious amount of blood flow seen in drilling the bone core *in vivo*. The placement of a layer of adhesive at the bone core hole base was sufficient to stabilize the blood flow and enable gluing of the bone cores with the OsStic adhesive. Remarkably given the difficulty of translating this novel bone core technique from *ex vivo* to *in vivo*, the bone cores were retained in place in both the adhesive and Tisseel groups for every animal at every timepoint. The justification for using Tisseel as a control was that it would retain the bone core in place, a necessary condition for the study approved by the ethical committee.

The Tisseel results (see Figure 6) at days 0 and 3, with low magnitudes and standard deviations, are consistent with the values reported in an earlier *ex-vivo* study (Procter, 2019). Similarly, low values are reported in a nerve repair study tensile test where only Tisseel was used (Childe et al., 2018). All pulled out bone cores were intact. From day 7, bone core breakage was seen as well as pull-out, which may be attributed to the early signs of woven bone formation seen in the cancellous compartment and the increasing failure strength and stiffness at this timepoint. At days 14 and 28, the Tisseel-bonded cores continued to gain in failure strength and stiffness which is consistent with the additional bone formation and maturation observed in the histology. At day 28, the bone core failure mode changed to screw stripping (4/8 samples) as the core and cortical shell had been fully osseointegrated. The general characteristic of low initial strength and then rapid strengthening were seen in a canine osteochondral fragment model (Keller et al., 1985) tensile load at failure increased 7-fold from 0.73 N/cm^2^ (σ 0.11 N/cm^2^) at 30 min post application to 5.1 N/cm^2^ (σ 0.88 N/cm^2^) at 4 days. By 7–8 days, only 2 out of 8 fibrin glued fragments could be separated at the original fracture line at loads of 5.8 and 10 N/cm^2^. They concluded that “the low initial mechanical strength in the fractures fixed with fibrin sealant demands a reliable immobilization to prevent displacement”. This has always been a barrier to the adoption of fibrin adhesives in musculoskeletal applications where surgeons need to ensure primary stabilization at surgery.

**FIGURE 10:**
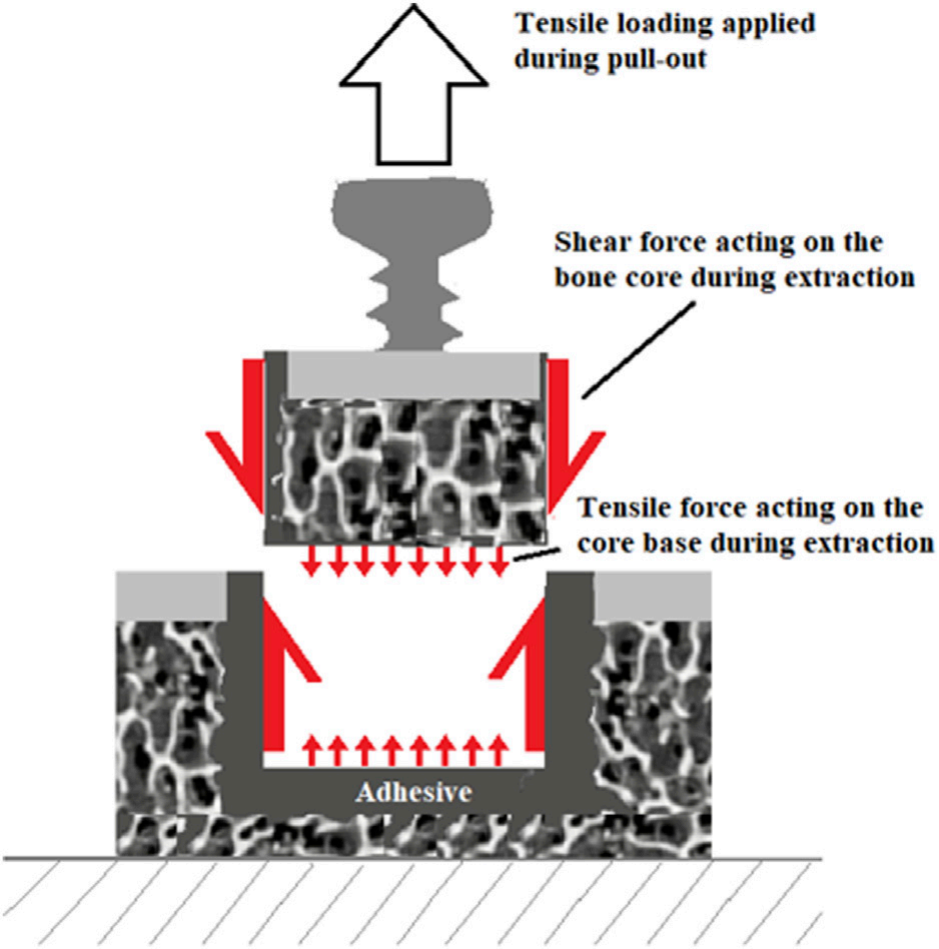
Schematic illustration of bone core loading during pull-out.

**FIGURE 11:**
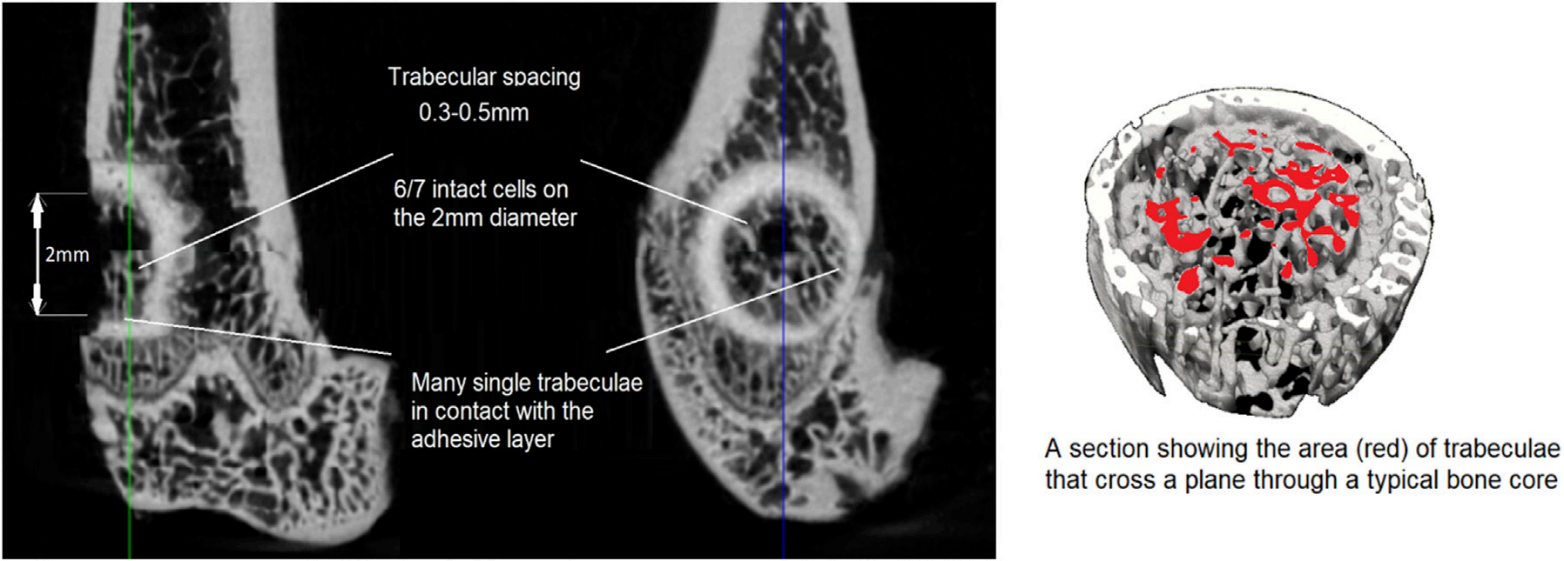
A typical adhesive bone core fracture surface and a section to illustrate a typical bone core trabecular cross section.

Turning to the adhesive bone core test results, no predicate study of sufficient similarity was found that would enable a meaningful comparison of the results obtained with the adhesive. While pull-out testing was used to have some measure of the holding power of the adhesive, it was not possible to determine exactly how the applied tensile loading distributed over the bone core surface. Generalizing, there will be shearing forces acting on the surface of the bone core cylinder as well as components of force due to the adhesive bonding from the end of the cylinder to the bottom of the core cavity, schematically illustrated in Figure 10. In both of the observed bone core failure modes, pull-out and breakage, showed the same characteristic failure curves: an initial small nonlinear portion followed by an approximately linear region; and then a very small ductile region followed by abrupt failure. In the bone core model, the load at failure is borne by a relatively small number of trabeculae, as illustrated in Figure 11. Indeed, it may be that only 15% or less of the cross section of a typical bone core carries the load at failure. To further develop this point, the cross section area is 3.142 mm^2^ (*π*d^2^/4, d = 2 mm) for the 2 mm diameter core so the effective load-bearing area is 3.142*0.15 mm^2^ = 0.4713 mm^2^. Assuming that the mean peak failure load at core breakage is 6.79 N (vide supra) and this is distributed uniformly across the effective trabecular load-bearing area, the corresponding stress would be 14.4 MPa. This is in the range reported for tensile failure of the bovine and human cancellous bone (Kaplan et al., 1985; Carter et al., 1980) and may be clinically relevant for bonding small osteochondral bone fragments.

The linear portion of the load displacement curves gave consistent stiffness values at each timepoint, despite the mixed failure modes, with stiffness rising to 31.56 N/mm at day 28. The literature is sparse when looking for comparable data; a comparable stiffness of 39.1 N/mm was estimated (Folwarczna et al., 2019, approximated from a maximum load 36.1 N/0.922 mm displacement at that load) in the young male Wistar rat proximal tibial metaphysis. Unlike the adhesive *mppf* curve, there is no dip seen in the adhesive stiffness values at day 7 which suggests that the transition from mechanical to biological fixation may reflect the quantity of new bone forming rather than the quality.

**FIGURE 12:**
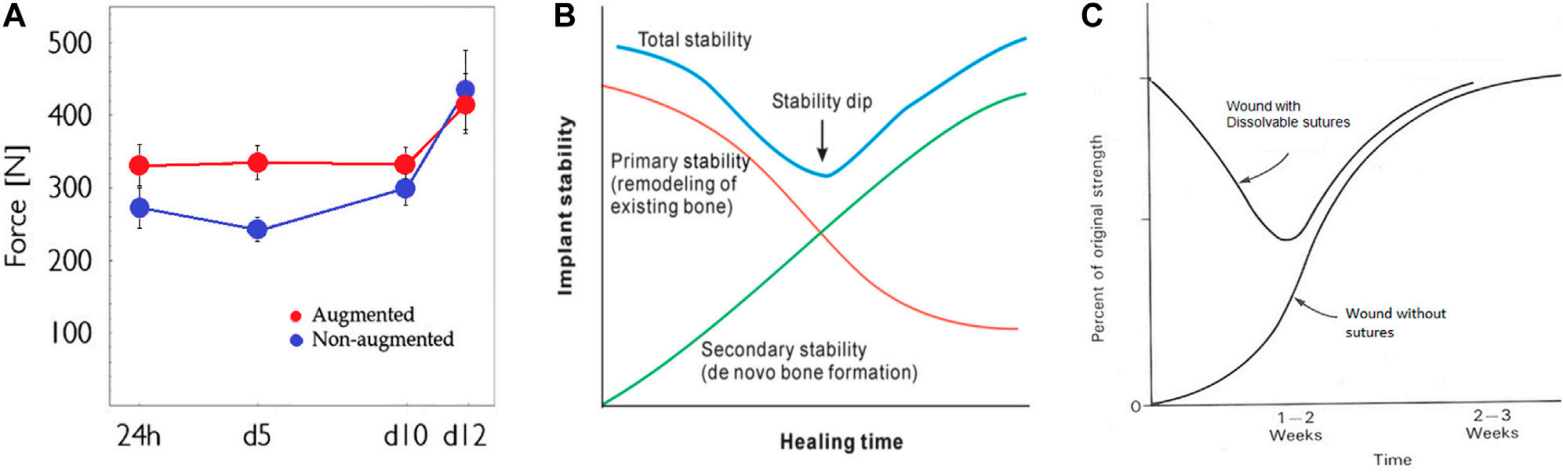
Implant stability dip during healing: **(A)** screw pull-out in the cancellous bone, **(B)** dental implant, and **(C)** soft tissue wound healing.

The overall shape of the adhesive *mppf* curve, see Figure 5, shows the initial value decreasing to a minimum with time and then recovering/healing at the latest time point is seen in other preclinical studies. For example, in a lapine augmented distal femoral titanium screw pull-out model, comparing calcium phosphate cement augmentation to an unaugmented screw, the control showed a minimum at day 5 with a higher initial value at 1 day and at day 10 (Larsson et al., 2012) see Figure 12A. In the dental implant field, an implant stability dip is observed between 1 and 8 weeks (Suzuki et al., 2013). This phenomenon is explained as the net effect of primary stability decreasing with time as existing bone remodels, and secondary stability increasing when *de novo* bone forms and osseointegrates the implant in the bone, see Figure 12B.

**FIGURE 13:**
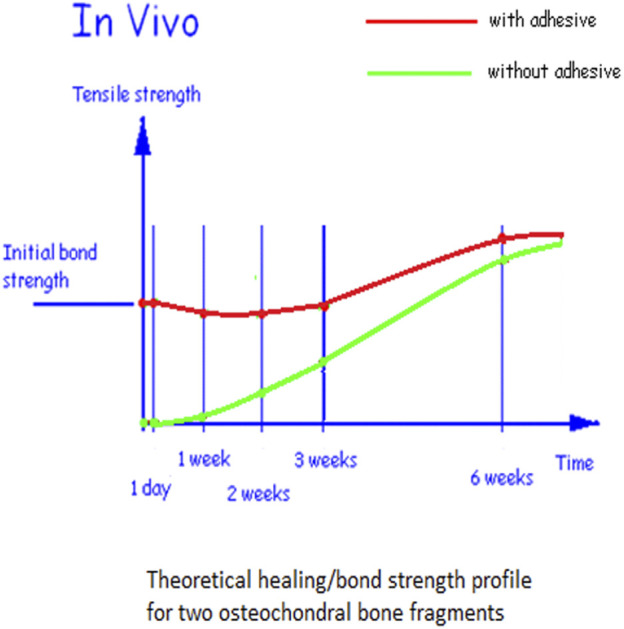
Hypothesis for the time course of tensile strength between two osseochondral fragments.

The development of healing strength in acute soft tissue wounds has been extensively studied and is similar to the dental implant stability time course. The initial mechanical stability conferred by resorbable sutures gives way to the increasing biological strength of the wound in a time scale of 1–2 weeks. The dip in strength is around 50% of the strength of intact skin (Hunt and Van Winkle., 1976), see Figure 12C. The possibility of a fixation stability dip in the development of osteochondral fragment bonding with and without an adhesive was already hypothesized (Procter, 2019), see Figure 13. The translation from purely mechanical fixation through to biological fixation with an intermediate transition dip is broadly similar in character in healing fixation of a bone screw, dental implant fixation, soft tissue repair, and now in the behavior of the present bone core model. This consistent result is in contrast to the literature (Cochran et al., 2020; Norton et al., 2020) reporting a similar TTCP+PSer-based adhesive which neither anticipates nor shows any strength dip.

The OsStic bone adhesive *mppf* curve, read in combination with the histology, shows that new bone formation occurs through the adhesive layer, which does not appear to form a barrier to normal healing, and at no time the initial bonding effect is entirely lost whilst reducing to half the initial strength at day 7 and then recovering the initial level by day 28. The 28 day histology shows that 40% of the adhesive in the cancellous compartment and 10% in the cortical compartment have remodeled so it is the newly formed cancellous bone that accounts for most of the recovery in *mppf*. The 42 day histology shows that this remodeling of the adhesive has continued, as now, 70% of the adhesive in the cancellous compartment and 50% in the cortical compartment have remodeled and the newly formed bone is equivalent to the original bone in quality and form at the osteotomy site. The excess adhesive that was outside the bone envelope has continued to be removed without any new bone formation. Forming the right bone only in the right place suggests that the adhesive is cell instructive and is a clinically desirable feature that the bone adhesive only forms bones where the operator intends. From histology and the biomechanical results, the rate of remodeling and replacement of the adhesive appears to be relatively fast. In a recent study (Lodoso-Torricilla et al., 2019), a rat retrograde distal femoral defect (2.5 mm Ø x 5 mm depth) model filled with calcium phosphate cement (∼total vol 24 mm^3^) found that 80% of the calcium phosphate cement remained for 8 weeks. Note that this is ∼3 times the volume filled in the present model and is cylindrical in form rather than a 0.4 mm layer on the surface of a cylindrical form.

The differentiated healing process in the cancellous bone proposed by Han et al., 2015 consisted of 5 stages: hematoma cell stage (1 day), proliferation (5 day), woven bone formation (14 day), lamellar bone formation (21 day), and finally the bone remodeling stage (42 day). This is consistent with the histological findings in the present study. Sandberg and Aspenberg (Sandberg and Aspenberg, 2016) described this as “intertrabecular” bone formation that was powered by locally available stem cells. They noted that this healing can be very rapid: “in rodents, a drill hole in the cancellous bone can be filled with new bone tissue in less than a week.” The early healing effects in the bone core model in the present study are consistent with this observation.

Reflecting on the sequence of events demonstrated after implantation of the bone core/adhesive, the following were determined: 1) initial adhesive fixation in a wet and fatty field by a chemical bonding process, 2) maintenance of the bone core fragment position during bone healing with no migration or displacement being observed out to 42 days, 3) new bone formation within the adhesive and new bone bridges in the existing bone where adhesive has remodeled in both cortical and cancellous compartments, 4) at 28 days, the tensile pull-out force returned to initial values from day zero and bone core breakage occurred in 5/8 animals. Histology showed that this effect is largely the new cancellous bone formation which has replaced 40% of the adhesive, and 5) at 42 days, the adhesive has been replaced by bone of equivalent type and quality (70% in cancellous and 50% in cortical compartments), and excess adhesive outside the bone envelope was removed, signifying that the adhesive is “cell instructive.”

Overall, the bone core *in vivo* model has worked sufficiently well in the present study to justify its further application in large animal models as a step toward first use in human indications.

### Limitations of the Study

The bone core model itself was novel and developed *ex vivo* and the present study is the first *in-vivo* evaluation. At the present time, there are no independent comparable cancellous/cortical bone models with which to benchmark the reported results. Interpretation of how the measured *mppf* values and stresses are distributed through the bone core and to the surrounding bone at failure is very limited in the present study, in particular, the trabecular load distribution at the core breakage site.

The rat is a common early animal model; however, the quality and density of the bone are not identical to those of larger animals, and in translating to humans, the effect of osteoporosis cannot easily be modeled. The surgical site preparation, adhesive mixing, amount of adhesive used in each animal, bone core positioning, harvesting and potting the bone samples, and mechanical test rig loading method all introduce variances. The effects of these are hard to quantify, and repeated *ex vivo* rehearsals were necessary to reduce their influence.

The excessive blood flow issue was not anticipated when preparing the bone core site. The adopted solution of sealing the base of each core with adhesive to reduce the flow worked as layering does not reduce adhesive layer strength. However, it points to an important issue to be addressed in further animal or human application.

The study term finishing at the 42 day time point (histologically) showed significant adhesive remodeling of bone; however, it remains to be confirmed that the entire adhesive is finally remodeled at a later time such as 90 days.

## Conclusion

The histological data show the OsStic adhesive harmonizes with the local bone biology in the murine bone core model with no signs of inflammation or adverse tissue effects throughout the study. Initial mechanical/chemical bond strength of 6.09 N is demonstrated with the bone core model at 4 h after surgery. The strength decreases at 7 days to a minimum of half the initial value, and then increases at 14 days to a value at 28 days that exceeds the initial fixation strength but with biologically incorporated bone cores. The healing of the cut bone core surfaces occurs through the adhesive layer with bone multicellular units replacing the adhesive with equivalent quality and quantity of the bone in both cortical and cancellous compartments up to 50% and 70%, respectively, at 42 days. The OsStic biomaterial demonstrated properties of adhesion, bioactivity, osseointegration, osteoconduction, and biodegradability, without inducing either local adverse effects or uncontrolled bone repair in both cortical and cancellous bone defects in the present rat model.

The research questions have been answered, and within the constraints of this animal study, the OsStic adhesive meets the requirements for a safe and effective bone adhesive as expressed in the literature. Its further preclinical evaluation in the animal fracture models is recommended as the next step in the path to first human applications.

## Data Availability

The datasets presented in this article are not readily available because the raw micro-CT and force test data are in file formats which require application-specific software to decode which we are unable to provide. We can provide selected micro-CT images and force displacement data on which the paper is based. Requests to access the datasets should be directed to philip.procter@angstrom.uu.se.

## Footnotes

1 Calculated from bone core Ø2 mm, osteotomy Ø2.8 mm (corresponds to trephine inner and outer diameters), depth of cut of 2 mm, and assuming a uniform layer 0.4 mm thick of adhesive.

## Abbreviations

*Mppf*, mean peak pull-out force; UC-VOI, under bone core volume of interest; CPC, calcium phosphate cement; Ø, diameter; PSer, phosphoserine; TCP, tricalcium phosphate; TTCP, tetracalcium phosphate; σ, standard deviation; cmN, centimeter newtons.
