# Supplementary material for: Gluing Living Bone Using a Biomimetic Bioadhesive: From Initial Cut to Final Healing
Source: Front Bioeng Biotechnol. 2021 Nov 8;9:728042. doi: 10.3389/fbioe.2021.728042 (PMC8606677; doi:10.3389/fbioe.2021.728042)
Supplement: Supplementary file 1 [file Table1.pdf]

Supplementary Tables

Table 1: Tisseel and Adhesive Histology summary days 3 and 7.

	Tisseel	Adhesive
Day3	Early-stage acute inflammation, dominated by plump macrophages, along with a slight fibroblastic reaction. Some Tisseel residue visible.	Noticeable cell proliferation, macrophages recruitment and binding to the adhesive material (AM). Very early signs of AM resorption. Activation of osteoblast precursors at the AM surface. Early signs of membranous mineralization.
Day 7	Infiltration of a large amount of macrophages along with fibrin exudate. Early fibroconnective tissue formation. Little activity at the cortical bone edges. Early signs of woven bone formation at the cancellous compartment. No direct bone-material contact.	Early stage of multinucleated giant cells (GCs) formation and hypertrophic macrophages resorbing the AM. No bone formation at the cortical bone edges. Peri-implant fibroblastic reaction and capillarisation with signs of osteogenic activity and initial and appositional bone formation.
	Fibroconnective reaction, acute inflammation, woven bone formation 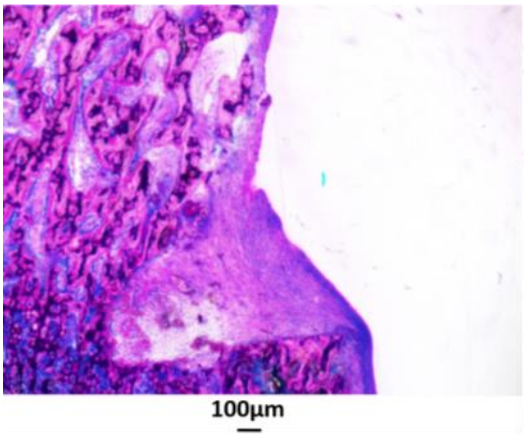	AM resorption. Active Phagocytic activity led by macrophages. 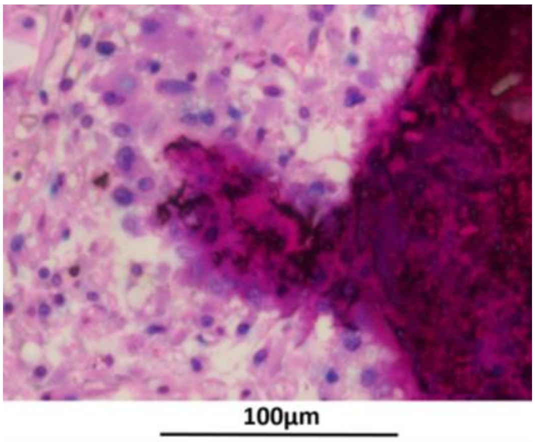

Table 2 Tisseel and Adhesive Histology summary day 14.

	Tisseel	Adhesive
Day 14	Evidence of active cellular resorption of the original cortical bone plug (CBP). Fibroconnective tissue mostly surrounding the residual CBP. New bone formation at the cortical and cancellous bone compartment forming osseous bridges with the CBP.<br>Tisseel material no longer visible.	Fibroblast and inflammatory cells infiltrate, mostly composed of GCs and osteoclasts creating channels into the AM with installation of intra-material BMUs (basic multicellular units). No bone formation at the cortical edges. At the subcortical level, bone coupling reflected by cell resorption activity at the AM surface and direct bone-material apposition started.
	Bone bridging the CBP. Large soft tissue presence.<br>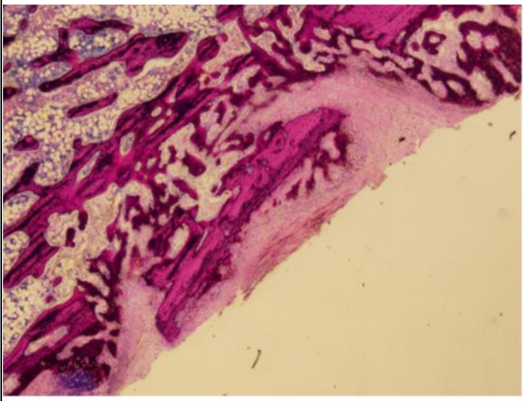<br>250µm	Bone-material coupling reflected by cell resorption activity (GCs/osteoclasts/macrophages) at the AM surface along with direct bone-material apposition<br>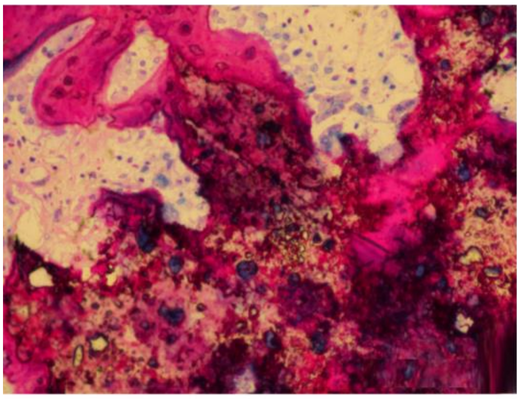<br>250µm

Table 3 Tisseel and Adhesive Histology summary day 28.

	Tisseel	Adhesive
Day 28	One rat analysed. Thick fibro-connective tissue mostly infiltrated with mononuclear cells. Some bone formation at the cortical and subcortical compartment along with hematopoietic bone marrow generation.	Fibrous encapsulation of AM present outside the cortical bone with no signs of bone outgrowth. ≈10% of the AM degraded at the cortical level. At subcortical level, resorption shaping the AM (material remodelling) was led by the GCs. Macrophages showed intracellular material. Intra-material vascularized BMUs at least 3 times larger than normal BMUs. ≈40 % of the AM degraded. Appositional bone growth and visible remodelling of the newly formed bone. Hematopoietic bone marrow generation including fat cells. No signs of local adverse effects.
	Some breakage of the bone trabeculae 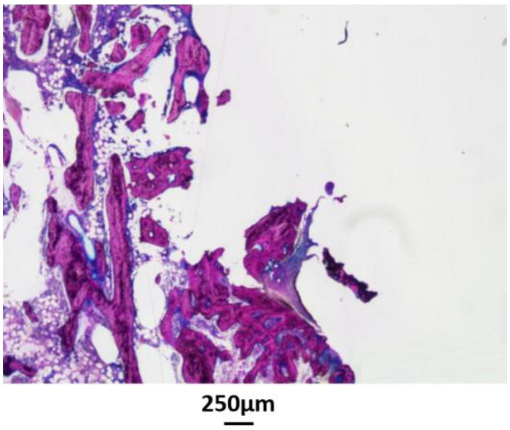	A basic multicellular unit (BMU) interfacing the AM and the newly formed bone 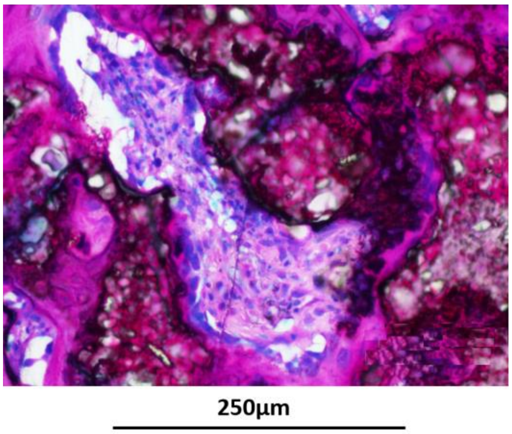

Table 4 Tisseel and Adhesive Histology summary day 42.

	Tisseel	Adhesive
Day 42	Advanced cortical bone healing but the anatomical shape not fully restored. Persisting fibrous tissue filling the residual cortical gap and depression. Restoration of cancellous bone achieved resulting in a normal cancellous bone. No signs of local side effects.<br>Cancellous bone regeneration with signs of trabecular maturity.<br>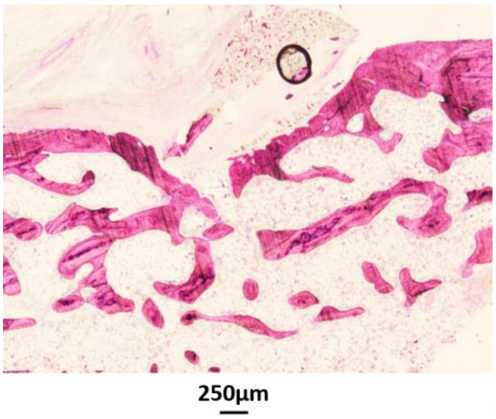	The AM maintained the CBP in position, as glue would be expected to do. No signs of abnormal bone growth outside of the defect, despite presence of excess of AM. The CBP lost osteocytes and vascular viability after implantation and displayed signs of resorption involving macrophages, GCs and osteoclasts. Active osteoblasts lining the outer CBP. BMUs visible at the cortical shell interface with the AM. Cortical interface showing a lamellar structure (interstitial lamellae) and early formation of haversian canals and osteonal structures. The CBP was osseointegrated. Approximately, 50% of the AM was resorbed at the cortical shell level. The cancellous bone was reformed and highly remodelled. Approximately, 70% of the AM was resorbed at the cancellous compartment level. No signs of deleterious local tissue effects.<br>See figures 3 and 4 in the paper, note these are from two different animals.
